# Axillary Lymph Node Dissection Can Be Omitted in Breast Cancer Patients With Mastectomy and False-Negative Frozen Section in Sentinel Lymph Node Biopsy

**DOI:** 10.3389/fonc.2022.869864

**Published:** 2022-04-14

**Authors:** Jing Si, Rong Guo, Huan Pan, Xiang Lu, Zhiqin Guo, Chao Han, Li Xue, Dan Xing, Wanxin Wu, Caiping Chen

**Affiliations:** ^1^ Department of Breast Disease, The First Hospital of Jiaxing, The Affiliated Hospital of Jiaxing University, Jiaxing, China; ^2^ Cancer Research Center, The First Hospital of Jiaxing, The Affiliated Hospital of Jiaxing University, Jiaxing, China; ^3^ Department of Breast Surgery, Breast Cancer Center of the Third Affiliated Hospital of Kunming Medical University, Cancer Hospital of Yunnan Province, Kunming, China; ^4^ Department of Central Laboratory, The First Hospital of Jiaxing, The Affiliated Hospital of Jiaxing University, Jiaxing, China; ^5^ Department of Pathology, The First Hospital of Jiaxing, The Affiliated Hospital of Jiaxing University, Jiaxing, China

**Keywords:** axillary lymph node dissection, sentinel lymph node biopsy, false-negative, frozen section, metastasis

## Abstract

**Background:**

The IBCSG 23-01 and AMAROS trials both reported that axillary lymph node dissection (ALND) did not change survival rates in breast cancer patients with positive nodes detected by sentinel lymph node biopsy (SLNB). The aim of this study was to determine whether breast cancer patients with mastectomy and false-negative frozen section (FS) in SLNB could forgo ALND.

**Materials and Methods:**

This was a retrospective study of cN0 patients diagnosed with primary invasive breast cancer treated by mastectomy and SLNB at our institute between January 2010 and December 2014. Patients with false-negative FS in SLNB were separated by the following management of axillary lymph node dissection in the non-ALND group (nonprocess or axillary radiation only) and ALND group (with or without radiation).

**Results:**

A total of 212 patients were included, 86 and 126 patients in the non-ALND and ALND groups, respectively. The positive rate of non-sentinel lymph nodes (SLNs) was 15.87% (20/126) in the ALND group. In multivariate analysis, we found that patients with larger tumor size (>2 cm) (OR, 1.989; *p* = 0.030) and multifocal lesions (OR, 3.542; *p* = 0.029) tended to receive ALND. The positivity of non-SLNs in the ALND group was associated with SLN macrometastasis (OR, 3.551; *p* = 0.043) and lymphovascular invasion (OR, 6.158; *p* = 0.003). Also, removing more SLNs (≥3) was related to negativity in non-SLNs (OR, 0.255; *p* = 0.016). After a median follow-up of 59.43 months, RFS and OS of the two groups were similar (*p* = 0.994 and 0.441). In subgroup analysis, we found that 97 patients who met the inclusive criteria of the IBCSG 23-01 trial had similar RFS and OS between the non-ALND and ALND groups (*p* = 0.856 and 0.298). The positive rate of non-SLNs was 9.62% (5/52). Also, in 174 patients who met the criteria of the AMAROS trial, RFS and OS in the non-ALND and ALND groups were similar (*p* = 0.930 and 0.616). The positive rate of non-SLNs was 18.27% (19/104).

**Conclusion:**

ALND can be carefully omitted in selected breast cancer patients with mastectomy and false-negative FS in SLNB. SLNB is relatively sufficient in the IBCSG 23-01-eligible patients, and axillary radiation was an effective option in the AMAROS-eligible patients.

## Introduction

Axillary lymph node (ALN) metastasis is one of the significant prognostic factors in early breast cancer. The status of ALN can affect postoperative treatment options, including adjuvant chemotherapy and adjuvant radiotherapy (RT) (1). Over the last two decades, sentinel lymph node biopsy (SLNB) is considered the standard of care for axillary staging in early breast cancer patients with cN0, with axillary lymph node dissection (ALND) only reserved for patients with positive SLNs (2, 3). Frozen section (FS) is the most widely used method in the intraoperative assessment of SLNs, which has the advantage to proceed immediately to ALND in patients with positive SLNs, avoiding a second axillary surgery (4, 5). However, the sensitivity of FS is limited, with a false-negative rate of up to approximately 20%, which may lead to patients being recalled for ALND (6–14).

In recent years, the role of ALND in the treatment of patients with a limited number of positive SLNs has been relegated. Several clinical trials have been conducted on omitting ALND in patients with SLN metastasis (15). The IBCSG 23-01 trial showed that ALND could be omitted in cT1-2 patients with SLN micrometastasis (16, 17). Subsequently, the American College of Surgeons Oncology Group (ACOSOG) Z0011 trial showed that ALND could be avoided without affecting recurrence rate or survival in patients with cT1-2 and 1-2-positive SLNs treated with breast conversing surgery (BCS) (18). Furthermore, the EORTC 10981-22023 AMAROS trial showed that axillary RT could be an alternative to ALND in patients with cT1-2 with no significant differences in recurrence rate or overall survival (19). Thus, with the results of these trials and the fact that ALND may lead to higher complications and poorer quality of life, the role of ALND in early breast cancer patients with positive SLNs is controversial, especially its contribution to survival.

Interestingly, we found that most patients in these trials received BCS. Although BCS is mostly performed in patients with early breast cancer, mastectomy is still inevitable due to various factors, such as tumor burden, tumor location, patient preference, etc. According to previous studies, approximately 70% of patients received mastectomy in China (20, 21). Thus, the aim of this study was to evaluate axillary management and clinical outcomes of cN0 early breast cancer patients treated with mastectomy and found to have false-negative FS in SLNB. Furthermore, the study aimed to validate the results of both the IBCSG 23-01 and AMAROS trials in our population and determine whether breast cancer patients with mastectomy and false-negative frozen section in SLNB could forgo ALND.

## Materials and Methods

We retrospectively reviewed the medical records of cN0 patients diagnosed with primary invasive breast cancer who were treated by mastectomy and SLNB at our institute between January 2010 and December 2014. Patients were excluded if: (1) patients were male; (2) neoadjuvant chemotherapy was received prior to surgery; (3) patients had bilateral breast cancer; or (4) patients had a history of breast cancer. The study was approved by the Ethics Committee of Jiaxing University.

Patients in this study received mastectomy and SLNB at our institution. SLNB was performed at the same time as mastectomy. Methyl blue method was used to identify SLNs. All patients were injected with approximately 2 ml of methyl blue in the subcutaneous layer around the tumor after anesthesia and were gently massaged for 5 min from the tumor to the ipsilateral axillary region to facilitate the transmission of methyl blue. Blue-stained SLNs were tested. Clinically suspicious nodes in the surgical field were also resected as SLNs. The number of SLNs for FS was decided by surgeons. Intraoperative FS assessment of SLNs was performed accordingly. SLNs more than 4 mm were bisected through long axis. Half of the nodes were embedded and frozen at −20°C. SLNs less than or equal to 4 mm were completely frozen. Sections were cut and stained with hematoxylin and eosin (H&E) for frozen examination. Histological assessment with hematoxylin–eosin staining performed postoperatively served as the gold standard. A positive SLN was defined as the presence of either micrometastasis (>200 cells or >0.2 mm, but <2.0 mm) or macrometastasis (>2.0 mm) identified on hematoxylin–eosin staining. The definition of false-negative FS in SLNB was that, in intraoperative FS assessment, SLNs were all negative, however, in postoperatively histological assessment, one or more SLNs were shown positive with micrometastasis or macrometastasis. Patients with false-negative FS in SLNB could either perform ALND or not according to surgeons’ choices. Levels I and II ALND were performed according to a standard ALND procedure in patients who underwent ALND. In the current article, patients with false-negative FS in SLNB were separated by the following management of axillary lymph node dissection in the non-ALND group (nonprocess or radiation only including axillary area) and ALND group (with or without radiation including axillary area). Immunohistochemistry (IHC) for ER and PR was performed, and cases with more than 1% were considered positive staining. HER2 positivity was defined as cases where IHC was 3+ or 2+ with fluorescence *in situ* hybridization (FISH) positivity. In addition, IHC for Ki67 was also performed and cases with more than 14% were considered positive staining.

Date and status at last follow-up were collected. Recurrence events were recorded as local–regional and distant. Time to recurrence and time to death were measured from date of surgery and censored at the date of last follow-up for event-free patients. Time to recurrence was censored at time of death. Loss to follow-up was defined as patients with less than 2-year follow-up after surgery.

The clinicopathological characteristics were compared between patients in the non-ALND group and ALND group using chi-square test for categorical variables. Time-to-event outcomes were estimated using Kaplan–Meier methods and were compared across groups using the log-rank test. Two-tailed *p*-values were adopted, and *p* < 0.05 was considered significant. All statistical analysis was performed using SPSS statistical software version 18.0 (IBM, Chicago, IL, USA).

## Results

A total of 1,470 cN0 patients who received mastectomy and SLNB were included, of which 14.42% (212/1470) had false-negative FS in SLNB. The median number of SLNs removed was 3, ranging from 1 to 12. In patients with false-negative FS, 86 (40.57%) and 126 (59.43%) were in the non-ALND and ALND groups, respectively. The positive rate of non-SLNs was 15.87% (20/126) in the ALND group. The baseline characteristics of the cohort with false-negative FS are shown in **Table 1**. We found that patients with larger tumor size (>2 cm) (*p* = 0.025), multiple number of foci (*p* = 0.017), and macrometastasis in SLNs (*p* = 0.045) had a tendency of receiving ALND. In multivariate analysis, patients with larger tumor size (>2 cm) (OR, 1.989; *p* = 0.030) and multifocal lesions (OR, 3.542; *p* = 0.029) had a tendency of receiving ALND (**Table 1**).

**Table 1:** Baseline characteristics of clinicopathological factors in cN0 patients who received mastectomy and had false-negative FS in SLNB.

Variables	Total	Non-ALND	ALND	Univariate	Multivariate
	= 212	= 86	*N* = 126	*p*-value	OR (95% CI), *p*-value
Age				0.563	
≤50	116	45	71		
>50	96	41	55		
Grade				0.243	
I	9	5	4		
II	155	66	89		
III	48	15	33		
Number of foci				0.017	
One	189	82	107		Ref
Multiple	23	4	19		3.542 (1.138–11.026), *p* = 0.029
Size on pathology				0.025	
≤2 cm	139	64	75		Ref
>2 cm	73	22	51		1.989 (1.070–3.697), *p* = 0.030
LVI				0.469	
No	156	61	95		
Yes	56	25	31		
Number of SLNs				0.312	
<3	70	25	45		
≥3	142	61	81		
SLN metastasis				0.045	
Micrometastasis	118	55	63		Ref
Macrometastasis	94	31	63		1.759 (0.981–3.154), *p* = 0.058
ER				0.227	
Negative	24	7	17		
Positive	188	79	109		
HER2				0.670	
Negative	173	69	104		
Positive	39	17	22		
Ki67				0.763	
≤14%	69	29	40		
>14%	143	57	86		

Moreover, we analyzed the factors associated with non-SLN positivity in the ALND group (**Table 2**). We found that the positivity of non-SLNs in the ALND group was associated with SLN macrometastasis (OR, 3.551; *p* = 0.043) and lymphovascular invasion (OR, 6.158; *p* = 0.003). Also, removing more SLNs (≥3) was related to negativity in non-SLNs in these patients (OR, 0.255, *p* = 0.016) (**Table 2**).

**Table 2:** Factors associated with positivity of non-SLNs in the ALND group.

Variables	Negative non-SLNs	Positive non-SLNs	Univariate	Multivariate
	*N* = 106	*N* = 20	*p*-value	OR (95% CI), *p*-value
Age			0.894	
≤50	60	11		
>50	46	9		
Grade			0.243	
I	3	1		
II	75	14		
III	28	5		
Number of foci				
One	89	18	0.489	
Multiple	17	2		
Size on pathology				
≤2 cm	62	13	0.586	
>2 cm	44	7		
LVI			0.004	
No	85	10		Ref
Yes	21	10		6.158 (1.865–20.338), *p* = 0.003
Number of SLNs			0.002	
<3	35	14		Ref
≥3	71	6		0.255 (0.084–0.773), *p* = 0.016
SLN metastasis			0.015	
Micrometastasis	58	5		Ref
Macrometastasis	48	15		3.551 (1.038–12.149), *p* = 0.043
ER			0.618	
Negative	15	2		
Positive	91	18		
HER2			0.338	
Negative	86	18		
Positive	20	2		
Ki67			0.855	
≤14%	34	6		
>14%	72	14		

We further analyzed the adjuvant therapy in the non-ALND and ALND groups. We found that the receipt of hormonal therapy (60.47% [52/86] vs. 53.97% [68/126], *p* = 0.349), adjuvant radiotherapy including axillary area (11.63% [10/86] vs. 12.70% [16/126], *p* = 0.816), and adjuvant chemotherapy (74.42% [64/86] vs. 78.57% [99/126], *p* = 0.481) was similar in the non-ALND and ALND groups.

**Figure 1:**
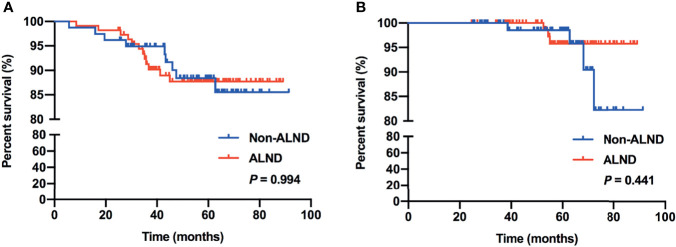
RFS **(A)** and OS **(B)** between the non-ALND and ALND groups.

In this cohort, 190 (89.62%) patients were included in the survival analysis. The median follow-up was 59.43 months, which was 4.95 years. Overall survival rate was 96.32% (183/190) for the total population in survival analysis. There were 20 (10.53%) recurrences: 5 local and regional; 13 distant; and 2 concurrent local and distant. The 5-year RFS rate and OS rate in the non-ALND and ALND groups are shown in **Table 3**. Moreover, Kaplan–Meier analysis showed that the RFS and OS of patients in the non-ALND and ALND groups were similar (*p* = 0.994 and 0.441, respectively) (**Figures 1A, B**).

**Table 3:** Five-year RFS rate and OS rate in the ALND and non-ALND groups.

	Total (*N* = 190)	Non-ALND group (*N* = 79)	ALND group (*N* = 111)	*p*-value
5-Year RFS rate	87.99%	88.41%	87.71%	0.799
5-Year OS rate	96.94%	98.53%	95.74%	0.208

**Figure 2:**
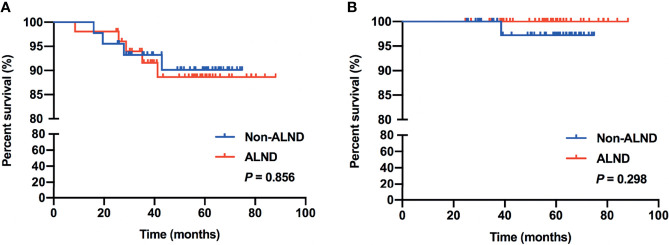
RFS **(A)** and OS **(B)** in patients who met the inclusive criteria of the IBCSG 23-01 trial.

**Figure 3:**
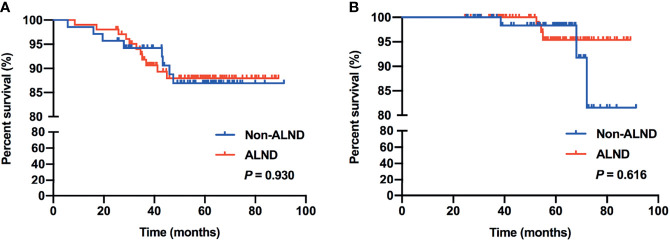
RFS **(A)** and OS **(B)** in patients who met the inclusive criteria of the AMAROS trial.

In subgroup analysis, we found that 97 patients met the inclusive criteria of the IBCSG 23-01 trial, of which, 46.39% (45/97) of patients were in the non-ALND group with 5 patients receiving radiation only, and 53.61% (52/97) of patients were in the ALND group, respectively. Similar RFS and OS were shown between the non-ALND and ALND groups (*p* = 0.856 and 0.298, respectively) (**Figures 2A, B**). The positive rate of non-SLNs was 9.62% (5/52) in the ALND group. In addition, we found 174 patients who met the criteria of the AMAROS trial, of which 40.23% (70/174) were in the non-ALND group with 9 receiving radiation only and 59.77% (104/174) were in the ALND group. We found that RFS and OS were similar between the non-ALND and ALND groups (*p* = 0.930 and 0.616, respectively) (**Figures 3A, B**). The positive rate of non-SLNs was 18.27% (19/104) in the ALND group.

## Discussion

SLNB is a minimally invasive procedure to determine the axillary status in patients with clinically negative lymph nodes. Currently, guidelines still recommend ALND if positive SLNs are found, except for patients who fit the criteria of several trials, including the ACOSOG Z0011 trial, IBCSG 23-01 trial, and EORTC 10981-22023 AMAROS trial, who can cautiously omit ALND (1, 15, 16, 18, 19). Therefore, accurate intraoperative SLN assessment is very important in order to avoid reoperation for patients with SLN metastasis. Among the various methods of intraoperative diagnosis of SLN status, FS is most commonly used. Previous studies showed that the false-negative rates of FS ranged from 13% to 43%, and the false-negative results occurred more frequently in cases with micrometastasis (5, 22–24). In our study, FS had a false-negative rate of 14.42% in patients who received mastectomy and SLNB, and 55.66% (118/212) were micrometastasis, which was consistent with previous studies. Our next focus is whether these patients with false-negative FS need to be recalled for ALND.

In recent years, several clinical trials have been focused on omitting ALND in patients with SLN metastasis (15). With the application of the criteria in these trials, ALND can be avoided in more patients with positive SLNs. However, most patients in these trials received BCS, including all the patients in the ACOSOG Z0011 trial, 91% of patients in the IBCSG 23-01 trial, and 82% of patients in the EORTC 10981-22023 AMAROS trial (16, 18, 19). Thus, with relatively solid evidence for patients who received BCS, the following questions remain to be resolved: (1) can patients who received mastectomy be managed the same way as BCS patients with regard to ALND and (2) is the use of intraoperative FS still needed if ALND can be omitted.

Previous studies have shown that omitting ALND is safe and has high survival rates in patients who received mastectomy with ≤2 positive SLNs (25, 26). Also, adding axillary radiotherapy can be a treatment option for patients with high-risk factors (25–27). In this current study, we retrospectively enrolled patients who received mastectomy with false-negative FS in SLNB. Several factors associated with the choice of ALND procedure and positive non-SLNs in patients who received ALND were found, including larger tumor size (>2 cm), multifocal lesions, lymphovascular invasion, SLN macrometastasis, and less-removed SLNs (<3). These were clinicopathological risk factors that could impact the decision of ALND or additional axillary radiotherapy, which were also consistent with previous studies of nomograms and models including these factors to evaluate the risk of further nodal involvement and might be used to select patients with positive SLNs in whom ALND may be safely omitted (28–30). In addition, we found that, with a median follow-up of 4.95 years, there were no significant differences in RFS and OS compared between the non-ALND and ALND group (*p* = 0.994 and 0.441, respectively). Also, in the IBCSG 23-01-eligible and AMAROS-eligible subgroups, survival outcomes were similar between non-ALND and ALND patients. Thus, with low axillary recurrence rate and relatively high survival outcome in patients with false-negative FS in SLNB without axillary clearance, we believe that omission of ALND may be considered in selected patients, especially with few clinicopathological high-risk factors.

On the other hand, under the circumstance that ALND can be omitted in selected patients, the need for intraoperative FS is controversial. FS remains the most common method of intraoperative assessment of SLNs with the advantage to proceed immediately to ALND in patients with positive SLNs avoiding further axillary surgery. However, with the possibility of omitting ALND in patients with positive SLNs, the role of FS is less important. Previous reports showed a significant decline in intraoperative assessment of SLNs from 69%–92% to 26%–45% before and after the disclosure of the ACOSOG Z0011 trial (31, 32). In addition, Bishop et al. reported that FS for SLNs dropped from 69% to 2% in their institute (33). Jorns et al. also reported a marked decline of FS from 74% to 25% pre-Z0011 and post-Z0011 (34). Nevertheless, if avoiding ALND in patients with positive SLNs is recommended, the indications regarding patients who meet the criteria of the above trials are still discordant, especially in patients who received mastectomy. What we believe we should admit is that, although ALND can be omitted in most patients who meet the criteria of certain trials, those with high-risk factors could have resulted in undertreatment. Thus, we suggest that intraoperative FS should not be routinely performed in all breast cancer patients; whereas, in selected high-risk patients who might be offered ALND after balancing the benefit from this procedure, intraoperative FS continues to be very useful in the intraoperative assessment of SLNs.

In addition, previous studies have been focused on increasing SLN detection rate in patients who need intraoperative assessment of SLNs. Several reports showed association between the method of SLN detection and sentinel lymph node identification, recommended combined method (radioactive tracer and blue dye), which could provide higher detection rate (35–37). Moreover, we found that removing more SLNs (≥3) was related to negativity in non-SLNs. Whereas, Dutta et al. indicated that no more than 4 SLNs should be removed because all patients with axillary metastasis were identified within the first 4 SLNs (38). Thus, we need to balance SLN detection rate and unnecessary SLN removal in clinical practice (39).

There were several limitations to this current study. Firstly, it was a retrospective study, which led to lower level of evidence. However, this study had a relatively large dataset with uniform inclusion and exclusion criteria. Secondly, few patients received axillary radiotherapy only in our institute, which provide limitations in our analysis. We analyzed survival outcomes in patients who received axillary radiotherapy only and patients who received ALND in the AMAROS-eligible subgroup, showing no significant difference. However, to avoid bias, we did not want to provide a conclusion based only on such a few cases of axillary radiotherapy. Further assessment is needed to accurately select patients in whom ALND can be safely avoided.

## Conclusion

In this study, we confirmed that there was no difference in patients’ survival regardless of additional ALND among early breast cancer patients who underwent mastectomy and false-negative intraoperative FS in SLNB. SLNB is relatively sufficient in patients who met the criteria of the IBCSG 23-01 trial, and axillary radiation was an effective option in patients who met the criteria of the AMAROS trial. Although further studies are needed, omission of ALND may be considered in selected patients. Thus, ALND can be carefully omitted in breast cancer patients with mastectomy and false-negative frozen section in SLNB.

## Data Availability Statement

The original contributions presented in the study are included in the article. Further inquiries can be directed to the corresponding author.

## Author Contributions

JS wrote the article. RG analyzed data. HP, XL, ZG, CH, LX, and DX collected data. WW and CC gave administration support. All authors listed have made a substantial, direct, and intellectual contribution to the work and approved it for publication.

## Funding

This work was supported by grants from the National Natural Science Foundation of China (Grant No. 81902674).

## Conflict of Interest

The authors declare that the research was conducted in the absence of any commercial or financial relationships that could be construed as a potential conflict of interest.

## Publisher’s Note

All claims expressed in this article are solely those of the authors and do not necessarily represent those of their affiliated organizations, or those of the publisher, the editors and the reviewers. Any product that may be evaluated in this article, or claim that may be made by its manufacturer, is not guaranteed or endorsed by the publisher.
